# Preparation and Properties of Nb^5+^-Doped BCZT-Based Ceramic Thick Films by Scraping Process

**DOI:** 10.3390/ma17174348

**Published:** 2024-09-02

**Authors:** Yang Zou, Bijun Fang, Xiaolong Lu, Shuai Zhang, Jianning Ding

**Affiliations:** 1 School of Materials Science and Engineering, Jiangsu Collaborative Innovation Center of Photovoltaic Science and Engineering, Jiangsu Province Cultivation Base for State Key Laboratory of Photovoltaic Science and Technology, National Experimental Demonstration Center for Materials Science and Engineering, Changzhou University, Changzhou 213164, China; s22010856091@smail.cczu.edu.cn (Y.Z.); xllu@cczu.edu.cn (X.L.); shuaizhang@cczu.edu.cn (S.Z.); 2 School of Mechanical Engineering, Yangzhou University, Yangzhou 225127, China

**Keywords:** BCZT-based ceramic thick films, film scraping process, sintering condition, conduction mechanism, low hysteresis strain

## Abstract

A bottleneck characterized by high strain and low hysteresis has constantly existed in the design process of piezoelectric actuators. In order to solve the problem that actuator materials cannot simultaneously exhibit large strain and low hysteresis under relatively high electric fields, Nb^5+^-doped 0.975(Ba_0.85_Ca_0.15_)[(Zr_0.1_Ti_0.9_)_0.999_Nb_0.001_]O_3_-0.025(Bi_0.5_Na_0.5_)ZrO_3_ (BCZTNb_0.001_-0.025BiNZ) ceramic thick films were prepared by a film scraping process combined with a solid-state twin crystal method, and the influence of sintering temperature was studied systematically. All BCZTNb_0.001_-0.025BiNZ ceramic thick films sintered at different sintering temperatures have a pure perovskite structure with multiphase coexistence, dense microstructure and typical dielectric relaxation behavior. The conduction mechanism of all samples at high temperatures is dominated by oxygen vacancies confirmed by linear fitting using the Arrhenius law. As the sintering temperature elevates, the grain size increases, inducing the improvement of dielectric, ferroelectric and field-induced strain performance. The 1325 °C sintered BCZTNb_0.001_-0.025BiNZ ceramic thick film has the lowest hysteresis (1.34%) and relatively large unipolar strain (0.104%) at 60 kV/cm, showing relatively large strain and nearly zero strain hysteresis compared with most previously reported lead-free piezoelectric ceramics and presenting favorable application prospects in the actuator field.

## 1. Introduction

At present, lead-based piezoelectric ceramics with excellent field-induced strain performance occupy a dominant position in the application field of actuators [1,2,3]. However, many countries around the world have advocated for environmental protection in recent years [4]. Due to the toxicity of PbO in raw materials, the preparation and application of lead-based ceramics are constrained, thus it is urgently necessary to develop lead-free ceramic materials with excellent electrostrain performance to gradually replace lead-based ceramics [4,5,6].

In 2009, Ren et al. initially proposed (Ba_0.85_Ca_0.15_)(Zr_0.1_Ti_0.9_)O_3_ (BCZT) lead-free piezoelectric ceramics with BaTiO_3_ as the matrix [7]. The unipolar strain (S_unipolar_) and the converse piezoelectric coefficient (d_33_^*^) values of BCZT ceramics were astonishingly 0.057% and 1140 pm/V under an electric field of 5 kV/cm, respectively, which attracted extensive attention from scholars both domestically and internationally. Unfortunately, the BCZT ceramics exhibited a high strain hysteresis (Hys) exceeding 20% at 5 kV/cm, which significantly restricted their applications in the actuator field. This limitation arises from the requirement for actuator materials to simultaneously possess high strain and low strain hysteresis [8,9]. Consequently, the significant challenge lies in enabling materials to maintain relatively large strain while simultaneously exhibiting extremely low strain hysteresis [9,10,11].

According to reports in the literature, the introduction of a second component to form a pseudo-binary solid solution in the piezo-materials can cause disorder in the composition and enhance the random field, disrupting the long-range ordered ferroelectric states within the materials and leading to the effective generation of polar nanoregions (PNRs) [12,13], which suppresses the strain hysteresis in materials due to their strong response to the external electric field [14,15]. For instance, Tian et al. incorporated the second component BaZrO_3_ into Na_0.52_K_0.48_Nb_0.9_Sb_0.1_O_3_ to form a pseudo-binary solid solution of (100 − x)Na_0.52_K_0.48_Nb_0.9_Sb_0.1_O_3_-xBaZrO_3_ [16]. As x increased from 0 to 4, the presence of PNRs caused the Hys value at 100 kV/cm to decrease from approximately 40% to 7.4%, while also exhibiting a relatively high strain of 0.2% [16]. Huang et al. introduced KNbO_3_ into BaTiO_3_ to create solid solution of (1 − x)BaTiO_3_-xKNbO_3_ [17]. Similarly driven by the presence of PNRs, the 0.94BaTiO_3_-0.06KNbO_3_ ceramics achieved an extremely low Hys of approximately 3% and a relatively high S_unipolar_ of approximately 0.10% at 80 kV/cm [17].

Additionally, it is widely recognized that soft donor doping can promote the rotation and switching of ferroelectric domains by creating cationic vacancies through high-valence ion substitution for low-valence ion, thereby reducing lattice stress and enhancing field-induced strain [18,19,20]. Simultaneously, the soft donor ion doping also leads to structural disorder and an enhanced random field, disrupting long-range ordered ferroelectric states and inducing the formation of PNRs within the piezo-materials, consequently reducing the strain hysteresis [19,21,22]. For example, Habib et al. reported the modification of 0.70Bi_1.03_FeO_3_-0.30BaTiO_3_ through A-site La^3+^ donor doping to obtain 0.70Bi_1.03_FeO_3_-0.30Ba_(1−x)_La_x_TiO_3_ ceramics [22]. As the x increased from 0 to 0.015, the S_unipolar_ value at 55 kV/cm rose from 0.150% to 0.188%, while the Hys value decreased from over 30% to approximately 20% [22]. Kang et al. modified 0.67BiFeO_3_-0.33BaTiO_3_ with B-site Sb^5+^ donor doping to obtain 0.67BiFe_1−x_Sb_x_O_3_-0.33BaTiO_3_ ceramics [19]. At 50 kV/cm, when x was 0.25%, the S_unipolar_ and Hys values of 0.67BiFe_99.75%_Sb_0.25%_O_3_-0.33BaTiO_3_ ceramics were 0.168% and 49%, respectively, exhibiting a significant improvement over the S_unipolar_ of 0.120% and the Hys of 59% in the 0.67BiFeO_3_-0.33BaTiO_3_ ceramics when x was 0 [19].

Therefore, in our previous research, (Bi_0.5_Na_0.5_)ZrO_3_ (BiNZ) was used as the secondary component to introduce into BCZT to form the solid solution (1 − x)(Ba_0.85_Ca_0.15_)(Zr_0.1_Ti_0.9_)O_3_-x(Bi_0.5_Na_0.5_)ZrO_3_ (BCZT-xBiNZ), where the orbital hybridization between the 6s orbital of Bi^3+^ and the 2p orbital of O^2−^ will result in high maximum polarization [23,24]. As we all know, the sintering temperature of preparing pure BCZT is comparably high (~1450 °C or above) [7], causing significant energy consumption [5]. Nevertheless, the low melting point characteristic of BiNZ can effectively reduce the sintering temperature and the energy consumption during the sintering process [23]. Via designing composition and tailoring sintering temperature, 1325 °C sintered BCZT-0.025BiNZ ceramics exhibited a higher relative density of 93.09% and optimal field-induced strain performance of S_unipolar_ 0.055% with low Hys of 6.55% at 20 kV/cm. In order to further improve the field-induced strain performance, B-site Nb^5+^ donor doping was undertaken to form 0.975(Ba_0.85_Ca_0.15_)[(Zr_0.1_Ti_0.9_)_1−x_Nb_x_]O_3_-0.025(Bi_0.5_Na_0.5_)ZrO_3_ (BCZTNb_x_-0.025BiNZ) ceramics. When x = 0.001, the S_unipolar_ value of BCZTNb_0.001_-0.025BiNZ ceramics sintered at 1325 °C reached 0.058% with a significantly reduced Hys of 3.65% at 20 kV/cm, presenting a marked improvement in field-induced strain performance.

As is widely recognized, traditional block ceramics are relatively thick, resulting in a greater number of defects and decreased dielectric breakdown strength under high external electric field compared with thinner ceramics [25]. While grinding and polishing manually or by machine can reduce the thickness of ceramics, extended preparation time is required and increased mechanical breakage is possible, which is not conducive to industrial production [25,26]. In 2020, Yan et al. successfully fabricated 0.75Bi_0.58_Na_0.42_TiO_3_-0.25SrTiO_3_ ceramic films with a thickness of ~0.04 mm using the film scraping process, which increased the maximum breakdown strength to 569 kV/cm [27]. Such results demonstrate that the film scraping process not only simplifies the fabrication process of thinner ceramics but also significantly enhances the maximum withstanding external electric field capability [25,26,27].

Furthermore, the preparation and processing of ceramic materials are closely intertwined with their structure and performance [23,28], among which the selection of sintering temperature is of paramount importance [23]. The sintering temperature directly influences the grain size and its uniformity in ceramics, consequently impacting their relative density and electrical properties [29,30]. It is worth noting that grain size is a significant factor influencing the strain performance of ceramics, as it affects the motion and switching of ferroelectric domains [19,31,32]. In the BCZT-based ceramics, the strain primarily originates from the switching of ferroelectric domains [33]. For example, Cai et al. investigated the sintering of BCZT ceramics in the temperature range of 1350 °C to 1500 °C and observed an increase in the grain size from 8.28 μm (1350 °C) to 44.37 μm (1500 °C) [33]. Correspondingly, the S_unipolar_ value of the BCZT ceramics increased from 0.086% (1350 °C) to 0.115% (1500 °C) at 20 kV/cm [33]. However, excessively high sintering temperatures are undesirable as they can lead to abnormal grain growth, increased grain size non-uniformity, greater surface pores in the microstructure, resulting in decreased relative density and consequently diminished material performance [31]. For example, Bijalwan et al. introduced 0.07 wt% CeO_2_ into BCZT, in which when the sintering temperature increased from 1275 °C to 1350 °C, the grain size increased from ~5 μm to ~13 μm, resulting in the increase in bulk density from ~5 g/cm^3^ to 5.64 g/cm^3^, maximum dielectric constant (ε_m_) at 1 kHz from ~2500 to ~4091 and piezoelectric constant (d_33_) from 50 pC/N to 507 pC/N after poling at 30 kV/cm; whereas further raising the sintering temperature to 1450 °C, the grain size of ceramics increased to over 25 μm, while bulk density, ε_m_ and d_33_ decreased to below 5.6 g/cm^3^, ~3500 and ~200 pC/N, respectively [31]. Hence, it is imperative to investigate the influence of sintering temperature on the structure and properties of ceramic materials.

Thus, this study employed the film scraping process to prepare the BCZTNb_0.001_-0.025BiNZ ceramic thick films, and the influence of sintering temperature on their structure and properties under relatively high external electric field was investigated. At 60 kV/cm, the relatively high S_unipolar_ (0.104%) and near-zero Hys (1.34%) were achieved in the BCZTNb_0.001_-0.025BiNZ ceramic thick film sintered at 1325 °C, exhibiting promising application prospects in actuator technology.

## 2. Experimental Procedure

### 2.1. Sample Preparation

**Figure 1:**
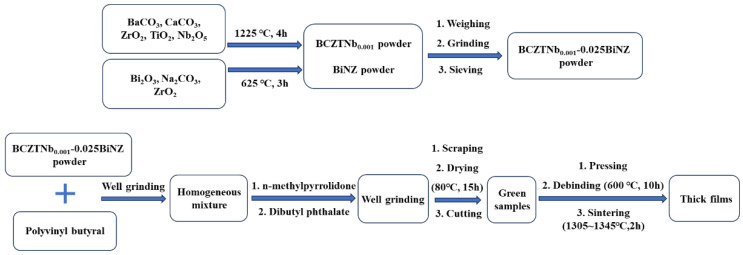
Schematic of preparing ceramic thick films by scraping process.

The schematic of preparing ceramic thick films by scraping process is shown in Figure 1. The 0.975(Ba_0.85_Ca_0.15_)[(Zr_0.1_Ti_0.9_)_0.999_Nb_0.001_]O_3_-0.025(Bi_0.5_Na_0.5_)ZrO_3_ (BCZTNb_0.001_-0.025BiNZ) ceramic thick films were prepared by a film scraping process [25,26], in which the BCZTNb_0.001_-0.025BiNZ powder was prepared by the solid-state twin crystal method, that was, the BCZTNb_0.001_ powder and BiNZ powder were synthesized separately [29]. All raw materials were commercially sold high purity oxides and carbonates from Sinopharm Group Chemical Reagent Co., Ltd. (Shanghai, China) without further processing and dried completely at 120 °C before preparation. Stoichiometrically weighed BaCO_3_, CaCO_3_, ZrO_2_, TiO_2_ and Nb_2_O_5_ were mixed and ground uniformly and calcined at 1225 °C for 4 h to prepare the BCZTNb_0.001_ powder. Similarly, thorough grinding of stoichiometrically weighed Bi_2_O_3_, Na_2_CO_3_ and ZrO_2_ was calcined at 625 °C for 3 h to obtain the BiNZ powder.

To prepare the BCZTNb_0.001_-0.025BiNZ ceramic thick films, accurately weighed stoichiometric BCZTNb_0.001_ powder and BiNZ powder were mixed and ground homogeneously in anhydrous ethanol, and then were sieved through a 150-mesh sieve. An appropriate quantity of polyvinyl butyral was added to the sieved mixture, and they were further ground until achieving uniformity. Subsequently, an appropriate quantity of n-methylpyrrolidone and dibutyl phthalate was added, followed by further grinding until the casting slurry was formed. The slurry was scraped onto a polyester release film with a scraper to form the thick films with a thickness of 0.75 mm. The scraped thick films were placed in an 80 °C oven for 15 h and then cut into circular film plates with a diameter of 12 mm. The organic compounds in film plates were separately removed at 600 °C for 10 h. The decarbonated films were sintered at 1305 °C–1345 °C for two hours to obtain ceramic thick films. Prior to sintering, the decarbonated films were covered with powder of the same composition in a covered alumina crucible to reduce the volatilization of Bi and Na. After sintering at different sintering temperatures, the BCZTNb_0.001_-0.025BiNZ ceramic thick films with a thickness of approximately 0.25 mm were obtained.

### 2.2. Materials Characterization

The crystal structure, phase structure, microstructure and grain size distribution of BCZTNb_0.001_-0.025BiNZ ceramic thick films were analyzed using X-ray diffraction method (Rigaku D/max-2500/PC, CuKα_1_, λ = 1.5406 Å, Japan), GSAS 1 software, JSM-IT100 scanning electron microscopy (SEM) and nano-measurement software, respectively. In order to further evaluate their electrical properties, the ceramic thick films were coated with silver paste on both sides and fired at 650 °C for 0.5 h. The dielectric properties from room temperature to 180 °C at 100 Hz–2 MHz and complex impedance spectra at high temperatures (390 °C–550 °C or 410 °C–550 °C) at 100 Hz–1 MHz were measured by Partulab HDMS-1000 system. The ferroelectric properties (P-E) at 60 kV/cm and 1 Hz, as well as the field-induced strain (S-E) properties at 60 kV/cm and 10 Hz, were tested by a ferroelectric analyzer (Precision LC II, Radiant Technologies Inc., Albuquerque, NM, USA). Before testing the ferroelectric and field-induced strain properties, the ceramic thick films were immersed in silicone oil at a temperature of 80 °C to 100 °C for 5 min to mitigate the risk of breakdown during the testing process.

## 3. Results and Discussion

### 3.1. Phase Structure and Refinement

**Figure 2:**
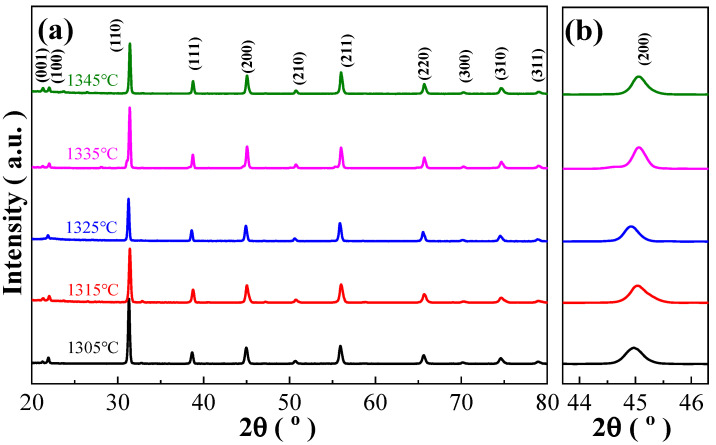
(**a**) XRD patterns of the BCZTNb_0.001_-0.025BiNZ ceramic thick films sintered at different sintering temperatures; (**b**) locally amplified XRD patterns of the BCZTNb_0.001_-0.025BiNZ ceramic thick films around (200) peak.

Figure 2a shows XRD patterns of the BCZTNb_0.001_-0.025BiNZ ceramic thick films sintered at 1305 °C to 1345 °C. All BCZTNb_0.001_-0.025BiNZ ceramic thick films exhibit a pure perovskite phase, indicating the complete integration of Nb^5+^ ion into the crystal lattice of BCZT-0.025BiNZ and forming a stable solid solution. Figure 2b shows locally amplified XRD patterns of the BCZTNb_0.001_-0.025BiNZ films, clearly demonstrating that there is no splitting in the (200) diffraction peak. Whereas the {100} diffraction reflection presents splitting into both the (100) and (001) peaks as evidently shown in Figure 2a, and the (200) peak displays broadening although without obvious splitting. Such phenomena suggest multiple phases coexist within the BCZTNb_0.001_-0.025BiNZ ceramic thick films [29], and the structure can generally be regarded as a pseudo-cubic structure [34,35].

**Figure 3:**
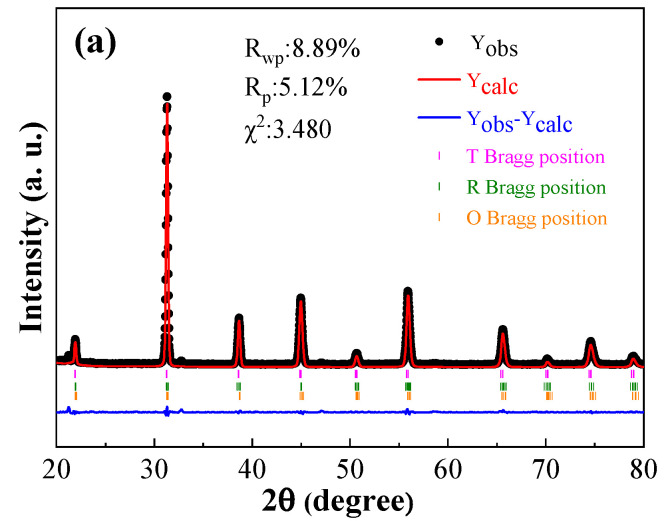

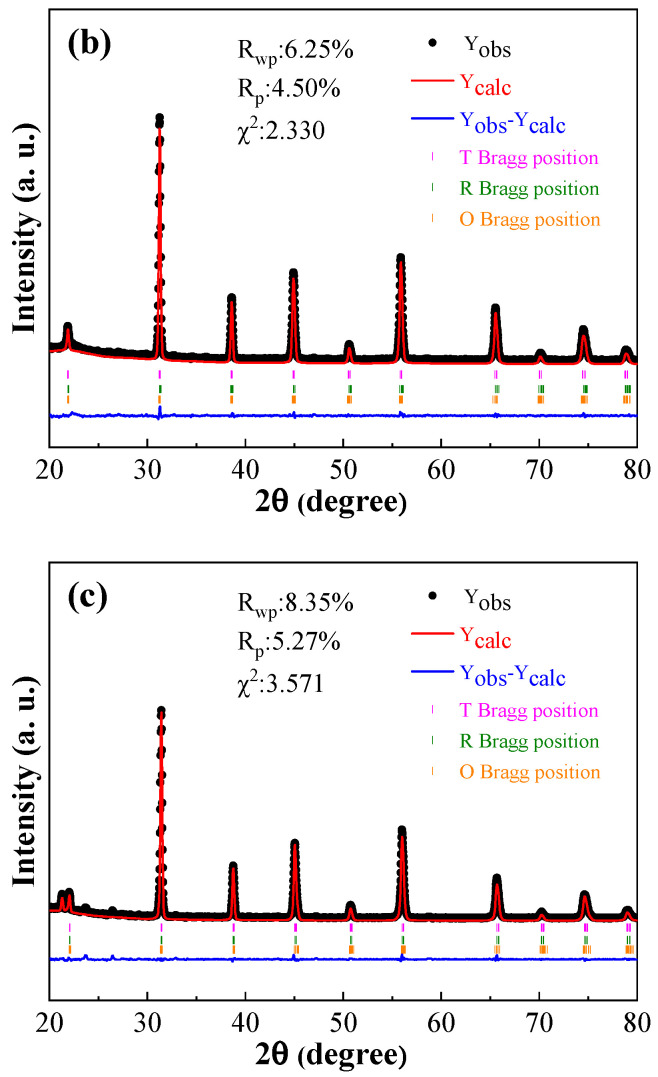
XRD Rietveld refinement results of the BCZTNb_0.001_-0.025BiNZ ceramic thick films sintered at different sintering temperatures: (**a**) 1305 °C; (**b**) 1325 °C; (**c**) 1345 °C.

Detailed multiple phases coexistence was analyzed by XRD Rietveld refinement as shown in Figure 3 of the BCZTNb_0.001_-0.025BiNZ ceramic thick films sintered at different sintering temperatures. The fitted R_wp_ and χ^2^ values for all ceramic thick films are below 8.9% and 3.6%, respectively, indicating a high degree of accuracy in the fitting results, and then, the XRD Rietveld refinement parameters are presented in Table 1. From analyzing Figure 3 and Table 1, it is evident that all ceramic thick films exhibit the presence of tetragonal, rhombohedral and orthorhombic phases simultaneously, presenting a similar phase structure to the pure BCZT ceramics reported by Ren et al. [7]. Notably, as the sintering temperature increases from 1305 °C to 1345 °C, the content of rhombohedral phase R3m increases from 8.17% (1305 °C) to 30.51% (1345 °C), demonstrating a pronounced upward trend. The tetragonal phase P4mm content in the 1305 °C and 1325 °C sintered films is comparable (~27%), which decreases significantly to 5.49% with further elevation of the sintering temperature to 1345 °C. With the increase in sintering temperature, the content of the orthorhombic phase Amm2 decreases apparently to 46.18% at the sintering temperature of 1325 °C, and then increases recoverably to a comparable value (~64%). In all ceramic thick films, the phase content of the orthorhombic phase obtained by Rietveld refinement is the highest, which is in line with the coexistence of multiple phases obtained from XRD, and the films can be viewed as a pseudo-cubic structure [34,35].

### 3.2. SEM Images and Grain Size Distribution

**Figure 4:**
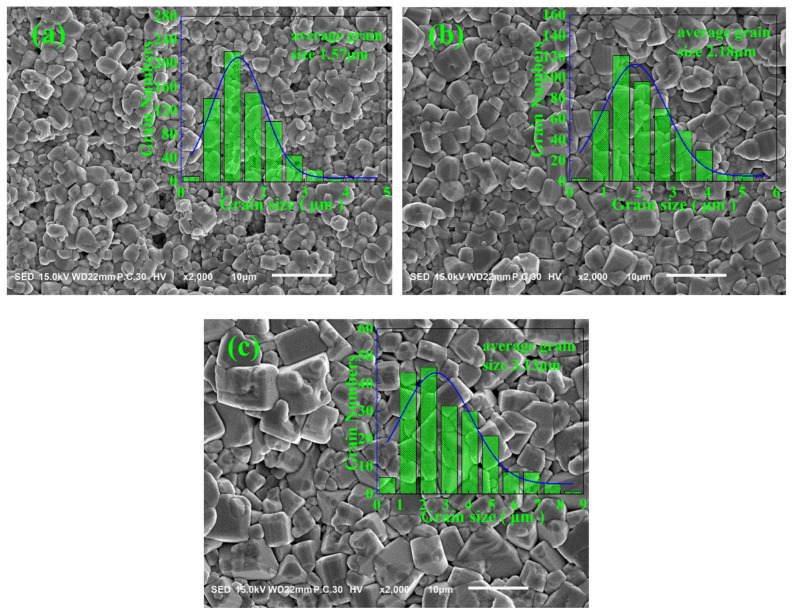
SEM images and grain size statistics (insets) of the BCZTNb_0.001_-0.025BiNZ ceramic thick films sintered at different sintering temperatures: (**a**) 1305 °C; (**b**) 1325 °C; (**c**) 1345 °C.

Figure 4 shows the SEM images and grain size distribution statistics of the BCZTNb_0.001_-0.025BiNZ ceramic thick films sintered at different sintering temperatures. The grain boundaries of all ceramic thick films are clear, in which the microstructure is dense and has relatively few surface pores. Additionally, the majority of grains are irregular polygons with a minority being nearly circular shape grains, indicating that the solid-state sintering is the primary mechanism during the sintering process, while liquid-phase sintering plays a secondary role [23,29,36]. As the sintering temperature rises, the grain size exhibits a nearly linear increase, and the average grain size of films reach 1.57 μm (1305 °C), 2.18 μm (1325 °C) and 3.13 μm (1345 °C), respectively. In comparison with the grain size of 8.28 μm for the pure BCZT ceramics sintered at 1350 °C reported by Cai et al. [33], the grain size of all ceramic thick film is remarkably decreased, suggesting that the introduction of a second component BiNZ restrains grain growth. When sintered at the lower temperature of 1305 °C, the ceramic thick film displays a smaller grain size with excellent grain distribution, whereas the grain growth is not sufficient, resulting in few surface pores and a lower density. Subsequently, at a higher sintering temperature of 1325 °C, grain growth becomes sufficient while still maintaining good grain distribution uniformity, leading to a denser structure without noticeable surface pores. However, as the sintering temperature is further increased to 1345 °C, the excessive high sintering temperature causes some grains to grow abnormally, resulting in a decrease in the uniformity of grain size. Overall, at an appropriate sintering temperature, the BCZTNb_0.001_-0.025BiNZ ceramic thick films can obtain dense microstructure and high density [25,26]. Additionally, compared with the higher sintering temperature (~1450 °C or above) for preparing pure BCZT ceramics [7], the BCZTNb_0.001_-0.025BiNZ ceramic thick films achieve low-temperature densified sintering at ~1325 °C, greatly reducing energy consumption.

### 3.3. Dielectric Properties and Dielectric Response Fitting

**Figure 5:**
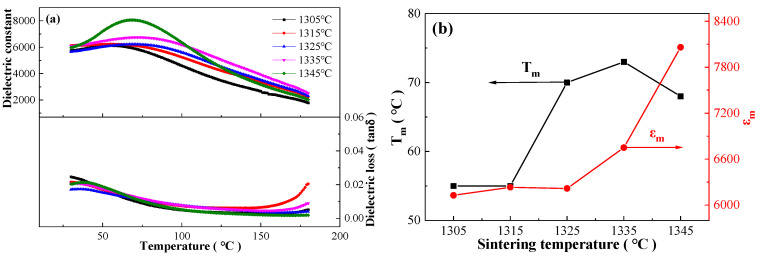
(**a**) Dielectric performance-temperature curves of the BCZTNb_0.001_-0.025BiNZ ceramic thick films sintered at different sintering temperatures at 10 kHz; (**b**) curves of T_m_ and ε_m_ with sintering temperature at 10 kHz.

Figure 5a,b shows dielectric properties versus temperature relationship of the BCZTNb_0.001_-0.025BiNZ ceramic thick films sintered at different sintering temperatures, as well as curves of the temperature of maximum dielectric constant (T_m_) and the maximum dielectric constant (ε_m_) with sintering temperature at 10 kHz, respectively. All ceramic thick films exhibit a broad dielectric peak, and dielectric constant tends to increase accompanied by the variation of T_m_ with elevating sintering temperature, indicating relaxor ferroelectrics characteristic. By further analyzing the two charts, it is observed that T_m_ initially increases and then decreases with the rising sintering temperature, in which the maximum T_m_ value of 73 °C is achieved in the 1335 °C sintered ceramic film. Additionally, the ε_m_ value fluctuates around ~6200 between sintering temperature of 1305 °C–1325 °C, which increases rapidly with further increasing sintering temperature, and reaches the maximum value of 8059.5 in the 1345 °C sintered ceramic film. For comparison, the ε_m_ and T_m_ values of the 1450 °C sintered pure BCZT ceramics are ~18,000 and ~93 °C, respectively, reported by Ren et al. [7]. Furthermore, the rapid increase in dielectric properties with elevating sintering temperature can be mainly attributed to the relationship between the dielectric properties of ceramic materials and grain size, in which larger grain size facilitates domain wall movement, resulting in a higher dielectric constant [31,32,37]. This finding aligns with the conclusion drawn from Figure 4. Furthermore, all BCZTNb_0.001_-0.025BiNZ ceramic thick films exhibit a rather low dielectric loss (<0.03), which is especially suitable for electronic industry application.

**Figure 6:**
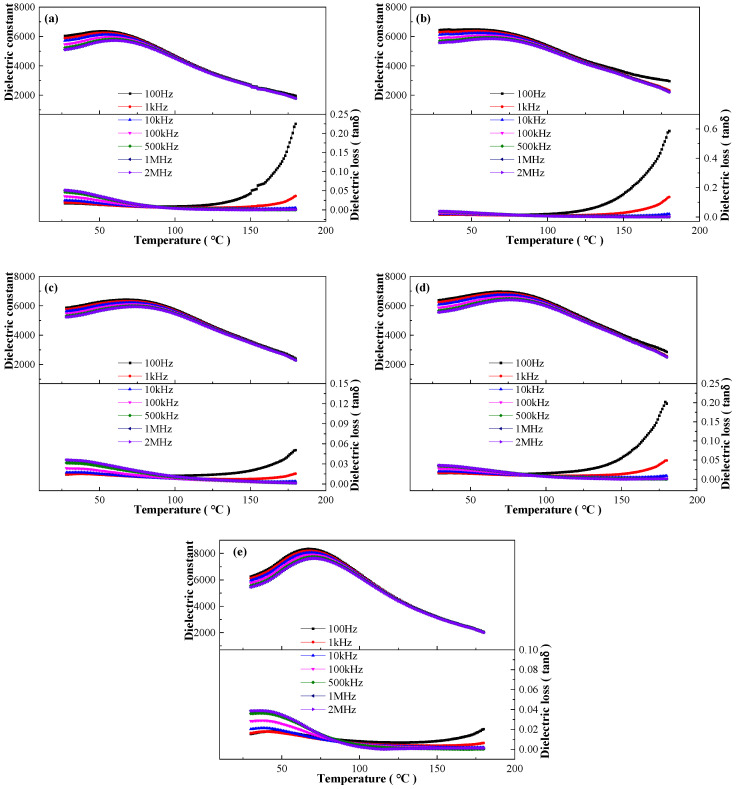
Dielectric performance-temperature curves at several frequencies of the BCZTNb_0.001_-0.025BiNZ ceramic thick films sintered at different sintering temperatures: (**a**) 1305 °C; (**b**) 1315 °C; (**c**) 1325 °C; (**d**) 1335 °C; (**e**) 1345 °C.

Figure 6 shows the effect of frequency on dielectric properties of the BCZTNb_0.001_-0.025BiNZ ceramic thick films sintered at different sintering temperatures to disclose the relaxor ferroelectrics nature. All ceramic thick films display pronounced frequency dispersion, especially below and around the T_m_ temperatures, and all dielectric peaks exhibit a broadening characteristic as compared with the sharpening dielectric peak of the normal ferroelectrics such as pure BaTiO_3_ [17], indicating the typical relaxor ferroelectrics behavior [10,38]. With the increase in sintering temperature, width of dielectric peak, dielectric constant and loss tangent, T_m_ and ε_m_ values change accordingly.

At high testing temperatures, all ceramic thick films exhibit a significant increase in dielectric loss particularly at low frequency 100 Hz, possibly attributed to the oxygen vacancy conduction resulting from the evaporation of some metals (Bi and Na) and the heterovalent substitution of Bi^3+^ at the A-site and Nb^5+^ at the B-site within the perovskite structure [39]. The defect equations that generate oxygen vacancies are as follows [40,41]:

(1) $$4{Na}_{Na}^{\times }+{2O}_{O}^{\times }\to 4Na↑+O_{2}↑+4V_{Na}^{’}+{2V}_{O}^{\cdot \cdot }$$

(2) $$4{Bi}_{Bi}^{\times }+{6O}_{O}^{\times }\to 4Bi↑+3O_{2}↑+4V_{Bi}^{’’’}+{6V}_{O}^{\cdot \cdot }$$

(3) $${Bi}_{2}O_{3}\overset{3(BaCa)O\cdot (ZrTi)O_{2}}{\to }2{Bi}_{Ba/Ca}^{\cdot }+V_{Ba/ca}^{\prime \prime }+{3O}_{O}^{\times }+3(ZrTi)O_{2}$$

(4) $$2{Nb}_{2}O_{5}\overset{5(BaCa)O\cdot (ZrTi)O_{2}}{\to }4{Nb}_{Zr/Ti}^{\cdot }+V_{Zr/Ti}^{\prime \prime \prime \prime }+{10O}_{O}^{\times }+5(BaCa)O$$

This conduction mechanism belongs to thermally activated conduction with a thermal activation discontinuity, so there will be larger dielectric loss at high temperatures [42], which will be further analyzed in the following complex impedance spectra study.

**Figure 7:**
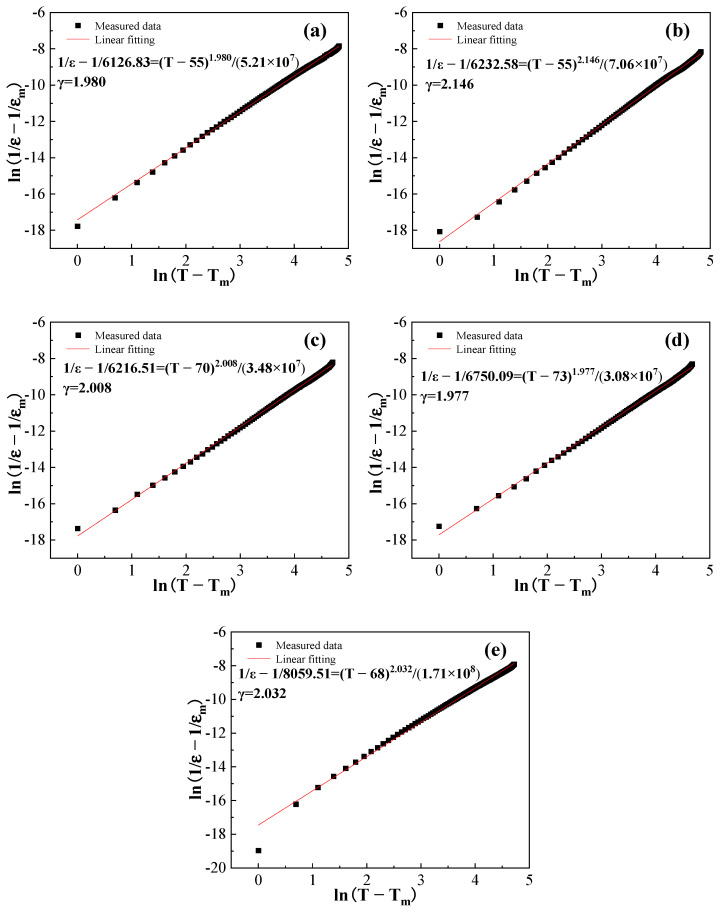
Dielectric exponential law fitting of the BCZTNb_0.001_-0.025BiNZ ceramic thick films sintered at different sintering temperatures using 10 kHz data: (**a**) 1305 °C; (**b**) 1315 °C; (**c**) 1325 °C; (**d**) 1335 °C; (**e**) 1345 °C.

The relaxation characteristic of the BCZTNb_0.001_-0.025BiNZ ceramic thick films sintered at various temperatures is further analyzed by the exponential law $\frac{1}{\varepsilon }-\frac{1}{\varepsilon_{m}}=\frac{{\left(T-T_{m}\right)}^{\gamma }}{C^{’}}$ fitting using the dielectric data at 10 kHz [38,41], where C’ is a constant and γ is the dispersion factor, and the results of exponential law fitting are shown in Figure 7. It is evident that all ceramic thick films exhibit nearly typical relaxor ferroelectrics nature and have a significantly large value of the dispersion factor γ of approximately 2, with specific values of 1.980 (1305 °C), 2.146 (1315 °C), 2.008 (1325 °C), 1.977 (1335 °C) and 2.032 (1345 °C), respectively. The above research indicates that the BCZTNb_0.001_-0.025BiNZ ceramic thick films possess a notably high level of relaxation degree.

### 3.4. Conduction Mechanism

**Figure 8:**
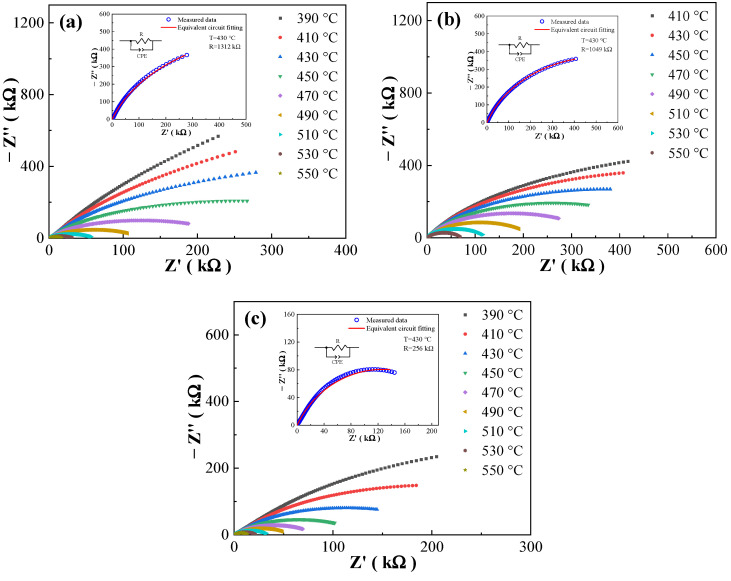
Z″ versus Z′ curves and the fitted equivalent circuits at 430 °C (insets) of the BCZTNb_0.001_-0.025BiNZ ceramic thick films sintered at different sintering temperatures: (**a**) 1305 °C; (**b**) 1325 °C; (**c**) 1345 °C.

In order to investigate the electrical conductivity and resistivity properties of materials, the complex impedance spectra of the BCZTNb_0.001_-0.025BiNZ ceramic thick films sintered at different sintering temperatures are measured and the Nyquist plots are shown in Figure 8, which is measured at elevated temperatures from 100 Hz to 1 MHz. It can be seen that all complex impedance spectra of all BCZTNb_0.001_-0.025BiNZ films measured at different testing temperatures present a single semicircle arc. The semicircles at lower testing temperatures are incomplete since the external energy is insufficient to stimulate the conduction process of ions and point defects within the ceramic thick films [12,25]. As the testing temperature increases, there is a gradual decrease in the diameter of semicircles in all films, indicating that the resistivity of samples is decreasing and the conductivity is increasing [25,43]. The fitted equivalent circuits of complex impedance spectra at 430 °C of respective ceramic thick films are shown in the insets of Figure 8, where R is the circuit resistance and CPE is the constant phase element. The volume resistivity of ceramic materials is jointly determined by the grain boundary resistance and grain resistance [26,43], whereas the complex impedance spectra of all samples can be fitted well with a single R and a single CPE connected in parallel, and the fitted results are in good agreement with the experimental data. The above results indicate that the resistance of all ceramic thick films is dominated by grain resistance. The fitted R value decreases gradually as the sintering temperature increases since as the grain size increases, the grain resistance decreases [26], which is consistent with the conclusion in Figure 4.

**Figure 9:**
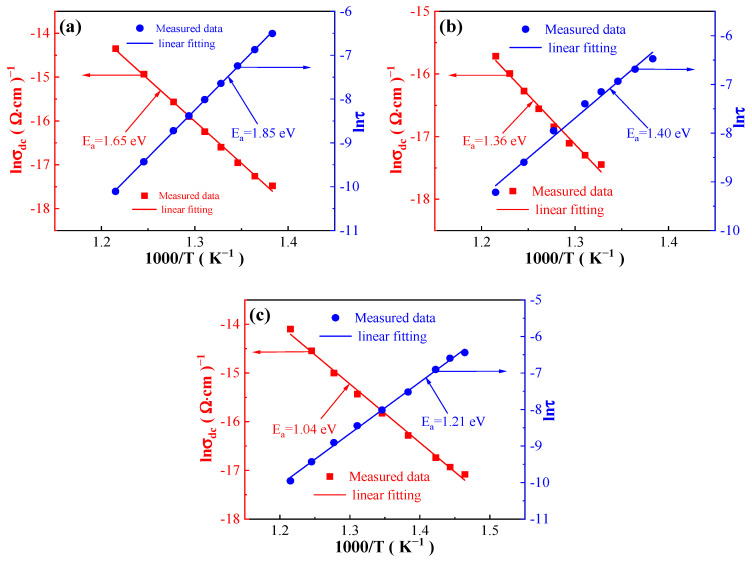
σ_dc_ and τ versus 1/T curves of the BCZTNb_0.001_-0.025BiNZ ceramic thick films sintered at different sintering temperatures: (**a**) 1305 °C; (**b**) 1325 °C; (**c**) 1345 °C.

In accordance with the Arrhenius law (Equations (5) and (6)) [40,44], where σ_dc_ is the DC conductivity, τ is the relaxation time, k_b_ is the Boltzmann constant, T is the thermodynamic temperature, E_a_ is the activation energy, σ_0_ and τ_0_ are constant, the conductivity activation energy and the relaxation activation energy can be calculated separately by linearly fitting the relationship between DC conductivity (σ_dc_) and relaxation time (τ) with temperature (T). Then, the conduction mechanism of the BCZTNb_0.001_-0.025BiNZ ceramic thick films sintered at different sintering temperatures can be studied, as shown in Figure 9.

(5) $$\sigma_{dc}=\sigma_{0}exp\left(-\frac{E_{a}}{{Tk}_{b}}\right)$$

(6) $$\tau =\tau_{0}exp\left(-\frac{E_{a}}{{Tk}_{b}}\right)$$

Based on the Arrhenius law linear fitting, the ceramic thick films sintered at different temperatures exhibit the conductivity activation energy of 1.65 eV (1305 °C), 1.36 eV (1325 °C) and 1.04 eV (1345 °C), while the relaxation activation energy is 1.85 eV (1305 °C), 1.40 eV (1325 °C) and 1.21 eV (1345 °C), respectively. Notably, the relaxation activation energy for each film exceeds the respective conductivity activation energy, which is caused by studying the thermal relaxation process from the perspectives of the dynamics of conductive rate and thermodynamics of conduction energy barrier [40]. The activation energy (E_a_) for oxygen vacancy conduction is reported ranging from 0.5 eV to 2 eV [43], and the conductivity activation energy as well as the relaxation activation energy for all ceramic thick films fall within this range (Figure 9). Consequently, the conduction mechanism of all BCZTNb_0.001_-0.025BiNZ ceramic thick films at high temperatures is dominated by oxygen vacancy conduction [43]. Such discussion confirms the speculated reason for the significant increase in dielectric loss, especially at low frequencies, under elevated test temperatures (Figure 6). The generation of oxygen vacancies is associated with the volatilization of certain metals (Na and Bi) and the heterovalent substitution of Bi^3+^ at the A-site and Nb^5+^ at the B-site of the perovskite structure [39] as illustrated in Equations (1)–(4) [40,41].

### 3.5. Ferroelectric and Field-Induced Strain Performance

**Figure 10:**
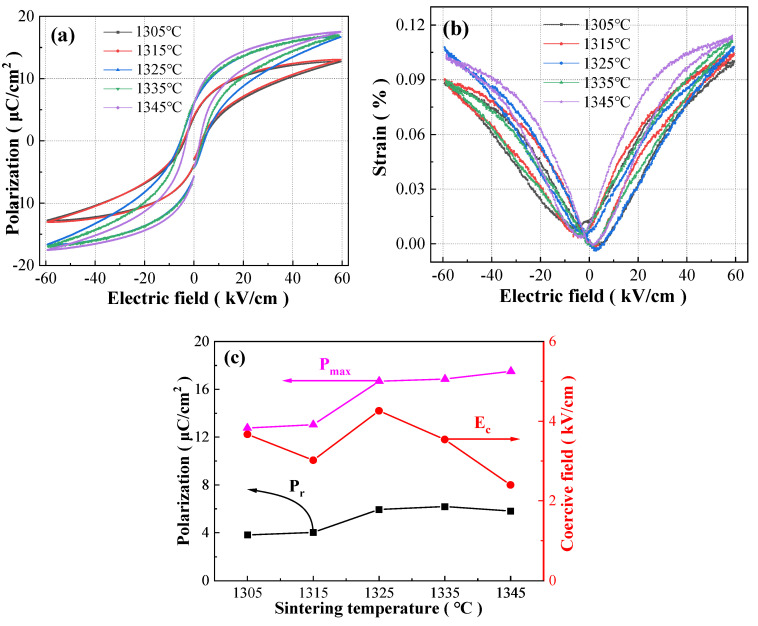
(**a**) P-E hysteresis loops at 60 kV/cm and 1 Hz and (**b**) bipolar S-E curves at 60 kV/cm and 10 Hz of the BCZTNb_0.001_-0.025BiNZ ceramic thick films sintered at different sintering temperatures; (**c**) curves of polarization and coercive field with sintering temperature of the BCZTNb_0.001_-0.025BiNZ ceramic thick films.

The P-E hysteresis loops (60 kV/cm and 1 Hz) and bipolar S-E curves (60 kV/cm and 10 Hz) of the BCZTNb_0.001_-0.025BiNZ ceramic thick films sintered at different temperatures are shown in Figure 10a,b. All ceramic thick films have relatively slender and symmetric P-E loops, and butterfly-like bipolar S-E curves with slight asymmetry, and the negative strain in the bipolar S-E curves can be ignored, which also indicates the relaxation characteristic of all BCZTNb_0.001_-0.025BiNZ films [4,12,35]. There is a slight non-closure phenomenon in the P-E loops, which is related to the periodic polarization of space charge field generated during sintering due to sublimination of Na and Bi metals and heterovalent substitution of Bi^3+^ and Nb^5+^ [39,45]. The bipolar S-E curves also show slight asymmetry, which is related to the existence of the internal biased field established by space charge field [14]. With the increase in sintering temperature, the P-E loops and S-E curves tend to augment accompanied by the improvement of ferroelectricity and electrostrain.

Figure 10c summarizes the variation in the maximum polarization (P_max_), the remnant polarization (P_r_) and the coercive field (E_c_) of the BCZTNb_0.001_-0.025BiNZ ceramic thick films with the sintering temperature. As the sintering temperature is raised from 1305 °C to 1345 °C, the P_max_ value of BCZTNb_0.001_-0.025BiNZ films shows an upward trend, increasing from 12.76 μC/cm^2^ to 17.51 μC/cm^2^. On the other hand, P_r_ initially increases and then decreases, and the largest P_r_ value of 6.19 μC/cm^2^ is obtained at a sintering temperature of 1335 °C. E_c_ fluctuates irregularly, presenting a decrease–increase–decrease trend, with the extremum minimum E_c_ value of 2.40 kV/cm at a sintering temperature of 1345 °C and the maximum E_c_ value of 4.26 kV/cm at a sintering temperature of 1325 °C. As a comparison, the P_r_ and E_c_ values of the 1450 °C sintered pure BCZT ceramics are ~15 μC/cm^2^ and 1.68 kV/cm, respectively, reported by Ren et al. [7]. The E_c_ value of BCZTNb_0.001_-0.025BiNZ ceramic films is rather small, which is easy for ferroelectric domain switching, and results in low hysteresis [19,20,33]. Comparatively speaking, the P_m_, P_r_ and E_c_ values of 0.65BiFeO_3_-0.35BaTiO_3_ at 60 kV/cm were 38.2 μC/cm^2^, 26 μC/cm^2^ and 20.7 kV/cm, respectively, reported by Yuan et al. [14], and the P_m_, P_r_ and E_c_ values of 0.67BiFe_99.75%_Sb_0.25%_O_3_-0.33BaTiO_3_ at 50 kV/cm were 33.23 μC/cm^2^, 26.93 μC/cm^2^ and ~28 kV/cm, respectively, reported by Kang et al. [19], whose E_c_ values are rather large. Additionally, the ferroelectric properties of ceramic thick films exhibit a certain improvement with the increase in sintering temperature, which is associated with the increase in grain size [29,31,33] and is consistent with the grain size variation trend observed in Figure 4.

**Figure 11:**
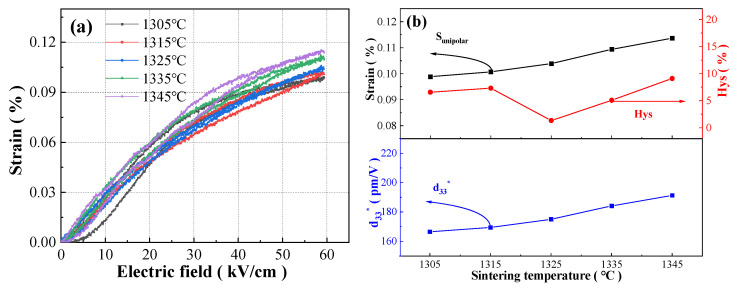
(**a**) Unipolar S-E curves at 60 kV/cm and 10 Hz of the BCZTNb_0.001_-0.025BiNZ ceramic thick films sintered at different sintering temperatures; (**b**) curves of S_unipolar_, d_33_^*^ and Hys with sintering temperature.

Figure 11 shows the unipolar S-E curves (60 kV/cm and 10 Hz), and variations in the unipolar strain S_unipolar_, the converse piezoelectric coefficient d_33_^*^ (d_33_^*^ = S_max_/E_max_ [46,47], S_max_ is the unipolar strain under the maximum electric field E_max_) and the strain hysteresis Hys (Hys = ΔS/S_max_ [46,47], ΔS is the difference in strain at half maximum field) with sintering temperature of the BCZTNb_0.001_-0.025BiNZ ceramic thick films sintered at different temperatures. The unipolar S-E curves of all ceramic thick films exhibit a similar slender shape, and the S_unipolar_ value shows an upward trend, increasing from 0.099% (1305 °C) to 0.114% (1345 °C) as the sintering temperature increases. The corresponding d_33_^*^ value also shows similar gradual increase with the elevation of sintering temperature, rising from 166.5 pm/V (1305 °C) to 191.3 pm/V (1345 °C). The increasing trend of S_unipolar_ and d_33_^*^ is also associated with the enlargement of grain size since the internal strain in the BCZT-based ceramics primarily originates from ferroelectric domain switching [33]. The larger grain size is more conducive to ferroelectric domain switching than the smaller grain size, so ceramic materials with larger grain size tend to have larger strain and d_33_^*^ [19,31,32,33], which is consistent with the conclusion drawn from Figure 4.

Furthermore, all BCZTNb_0.001_-0.025BiNZ ceramic thick films demonstrate very low Hys, each below 10%. The lowest Hys of 1.34% is achieved in the ceramic thick film sintered at 1325 °C accompanied by relatively high S_unipolar_ of 0.104% at 60 kV/cm, which can effectively meet the requirement of piezoelectric actuators for high strain and low hysteresis. Such low Hys can be attributed to the disordered internal structure and enhanced random field in the BCZTNb_0.001_-0.025BiNZ ceramic thick films resulting from the addition of a second component (BiNZ) and equivalent ion substitution, and A-site Bi^3+^ and B-site Nb^5+^ donor doping, which disrupts long-range ordered ferroelectric states and generates PNRs [12,13,19,21]. The intense response of PNRs to an external electric field can suppress strain hysteresis, thereby enabling BCZTNb_0.001_-0.025BiNZ to maintain lower strain hysteresis [14,15,17].

Table 2 summarizes the S_unipolar_ and Hys of the BCZTNb_0.001_-0.025BiNZ ceramic thick film sintered at 1325 °C and several other lead-free perovskite ceramic materials reported recently [8,9,17,19,22,47]. It can be observed that the BCZTNb_0.001_-0.025BiNZ ceramic thick film sintered at 1325 °C exhibits nearly zero Hys (1.34%) while it also maintains relatively large S_unipolar_ (0.104%) compared with other lead-free ceramic materials, where ceramics that have larger strain also possess an unacceptably higher Hys, suggesting excellent development prospects in the field of actuators.

## 4. Outlook

Presently, among the relatively effective approaches to improve electrostrain performance is the formation of PNRs within materials through doping cations or adding a second component [13,15,17]. In this article, the 1325 °C sintered BCZTNb_0.001_-0.025BiNZ ceramic thick film has attained an extremely low strain hysteresis (1.34%) at 60 kV/cm by generating PNRs; however, the unipolar strain (0.104%) still requires further enhancement. It is reported that the size and activity of PNRs exert an influence on the strain performance of ceramics. Small-sized and highly active PNRs will enable the material to achieve ultra-low strain hysteresis, but unfortunately, the corresponding strain will also decline [15]. Controlling the ferroelectric relaxor transition temperature is one of the known methodologies [38]. In the future, how to effectively regulate the size and activity of PNRs will constitute the key to attaining materials with extremely low hysteresis and high strain.

## 5. Conclusions

In this work, the BCZTNb_0.001_-0.025BiNZ ceramic thick films prepared by scraping process exhibit a pure perovskite structure, typical relaxation characteristic and high-temperature conduction mechanism of oxygen vacancies at different sintering temperatures. The scraping process simplifies the process of preparing thinner ceramic materials while significantly improving the external electric field resistance of ceramics. Due to the addition of a second component BiNZ with a low melting point, the ceramic thick film can be densely sintered at a low temperature of approximately 1325 °C. The grain size, dielectric, ferroelectric and field-induced strain properties of the BCZTNb_0.001_-0.025BiNZ ceramic thick films increase with elevating the sintering temperature. The 1325 °C sintered ceramic thick film has the lowest hysteresis (1.34%) and relatively high unipolar strain (0.104%) at 60 kV/cm, solving the problem that low hysteresis and large strain cannot coexist in the material, and showing good application prospects in the actuator field.

## Figures and Tables

**Table 1:** Space group, phase fraction, lattice constants of the BCZTNb_0.001_-0.025BiNZ ceramic thick films sintered at different sintering temperatures.

Sintering Temperature	Space Group	a (Å)	b (Å)	c (Å)	α = β = γ (°)	Fraction (%)
1305 °C	P4mm	4.019066	4.019066	4.015660	90	26.79
	R3m	4.010242	4.010242	4.010242	89.746	8.17
	Amm2	3.994396	5.674225	5.675882	90	65.04
1325 °C	P4mm	4.014551	4.014551	4.013084	90	27.81
	R3m	4.006793	4.006793	4.006793	89.845	26.01
	Amm2	4.001672	5.677942	5.689648	90	46.18
1345 °C	P4mm	4.012797	4.012797	4.008653	90	5.49
	R3m	4.011222	4.011222	4.011222	90.019	30.51
	Amm2	3.992369	5.675968	5.687695	90	64.00

**Table 2:** Comparison of strain properties of the BCZTNb_0.001_-0.025BiNZ ceramic thick film sintered at 1325 °C with some lead-free ceramics reported in the literature.

Compounds	S_unipolar_ (%)	Hys (%)	Electric Field (kV/cm)	Ref.
Ba_0.6_Sr_0.4_TiO_3_	0.07	4	60	[8]
0.91(K_0.48_Na_0.52_)Nb_0.965_Sb_0.035_-0.03Bi_0.5_ (K_0.18_Na_0.82_)_0.5_ZrO_3_-0.06BaZrO_3_	0.05	8	40	[9]
0.94BaTiO_3_-0.06KNbO_3_	0.10	3	80	[17]
0.67BiFe_99.75%_Sb_0.25%_O_3_-0.33BaTiO_3_	0.168	49	50	[19]
0.70Bi_1.03_FeO_3_-0.30Ba_0.985_La_0.015_TiO_3_	0.188	20	55	[22]
0.91Bi_0.5_Na_0.5_TiO_3_-0.07Ba(Zr_0.055_Ti_0.945_)O_3_-0.02(K_0.5_Na_0.5_)NbO_3_	0.31	33.5	65	[47]
BCZTNb_0.001_-0.025BiNZ	0.104	1.34	60	This work

## Data Availability

All data that support the findings of this study are included within the article, or available from the corresponding author upon request.

## Nomenclature

Amm2	an orthorhombic phase space group
P4mm	a tetragonal phase space group
R3m	a rhombohedral phase space group
BCZT	(Ba_0.85_Ca_0.15_)(Zr_0.1_Ti_0.9_)O_3_
BCZT-xBiNZ	(1 − x)(Ba_0.85_Ca_0.15_)(Zr_0.1_Ti_0.9_)O_3_-x(Bi_0.5_Na_0.5_)ZrO_3_
BCZTNb_x_-0.025BiNZ	0.975(Ba_0.85_Ca_0.15_)[(Zr_0.1_Ti_0.9_)_1−x_Nb_x_]O_3_-0.025(Bi_0.5_Na_0.5_)ZrO_3_
BCZTNb_0.001_-0.025BiNZ	0.975(Ba_0.85_Ca_0.15_)[(Zr_0.1_Ti_0.9_)_0.999_Nb_0.001_]O_3_-0.025(Bi_0.5_Na_0.5_)ZrO_3_
BiNZ	(Bi_0.5_Na_0.5_)ZrO_3_
CPE	constant phase element
C’	constant
d_33_	piezoelectric constant
d_33_^*^	converse piezoelectric coefficient
E_a_	activation energy
E_c_	coercive field
E_max_	maximum electric field
Hys	strain hysteresis
k_b_	Boltzmann constant
P_m_	maximum polarization
P-E	polarization-electric field hysteresis loop
PNRs	polar nanoregions
P_r_	remnant polarization
R	circuit resistance
R_p_	Rietveld refinement fitting reliability parameter
R_wp_	Rietveld refinement fitting reliability parameter
χ^2^	Rietveld refinement fitting reliability parameter
SEM	scanning electron microscope
S-E	strain-electric field loop
S_max_	unipolar strain under the maximum electric field
S_unipolar_	unipolar strain
T	temperature
T_m_	the temperature of maximum dielectric constant
XRD	X-ray diffraction
Z′	impedance real part
Z″	impedance imaginary part
ε_m_	maximum dielectric constant
γ	dispersion factor
σ_dc_	DC conductivity
σ_0_	constant
τ	relaxation time
τ_0_	constant
ΔS	difference in strain at half maximum field
